# Developing negative split pacing in endurance athletes: practical guidelines and training models

**DOI:** 10.3389/fphys.2026.1741125

**Published:** 2026-01-12

**Authors:** Gerasimos V. Grivas, Kousar Safari, Mohammad Hemmatinafar

**Affiliations:** 1 Physical Education and Sports, Division of Humanities and Political Sciences, Hellenic Naval Academy, Piraeus, Greece; 2 Department of Sport Sciences, Faculty of Education and Psychology, Shiraz University, Shiraz, Iran

**Keywords:** heart rate variability, lactate threshold, negative split, pacing strategy, VO_2_ kinetics, wearable technology

## Abstract

Negative split pacing—finishing the second half of a race faster than the first—is a hallmark of elite endurance performance and a key indicator of physiological control, fatigue resistance, and strategic pacing literacy. Rather than being an inherent talent, it represents a trainable skill integrating physiological, cognitive, and behavioral regulation. This mini review synthesizes evidence from performance analyses, physiological studies, and coaching practice to outline how negative split capacity can be systematically developed. We summarize training foundations, mesocycle design, and monitoring tools that support its acquisition. Mechanistically, successful negative splits are linked to improved lactate threshold (LT), VO_2_ kinetics, running economy (RE), central fatigue resistance, and pacing awareness. Practically, progression runs, split-pace long runs, and interval ladders promote these adaptations through controlled exposure to fatigue and deliberate perceptual feedback. A 6-week exemplar mesocycle illustrates how structured planning with pace anchors and feedback loops (HRV, GPS, session-RPE) refines pacing control and race execution. Psychological skills—including attentional focus, restraint, and adaptive decision-making—are essential to resist early surges and preserve finishing capacity. While negative split pacing may be constrained in short/middle-distance events, heat, altitude, or tactical racing, in endurance road events it offers a robust, learnable performance model. Overall, negative split pacing is not merely an outcome of elite talent but a trainable strategy rooted in physiology, cognition, and feedback-informed coaching. With deliberate practice and individualized monitoring, athletes across levels can enhance consistency, resilience, and race-day outcomes.

## 1 Introduction

Negative split pacing—the strategy of completing the second half of a race faster than the first—is widely regarded as an indicator of optimal endurance performance (Grivas, 2025a). Operationally, a negative split can be defined as completing the second half of a race at a mean pace approximately 1%–3% faster than the first, reflecting deliberate energy conservation early and controlled acceleration in the closing stages (Abbiss and Laursen, 2008; March et al., 2011; Hanley, 2015). Elite runners frequently adopt this strategy in championship settings and time trials, with historical examples spanning from Olympic marathons to world record attempts (Abbiss and Laursen, 2008; Hanley, 2015). Despite its association with superior performance in long-distance events, relatively few athletes consistently manage to implement negative split pacing effectively in practice (Renfree and St Clair Gibson, 2013).

Our previous work synthesized the physiological and psychological underpinnings of negative split performance, emphasizing the role of energy conservation, thermoregulation, perceptual regulation, and central fatigue resistance in facilitating this pacing pattern (Grivas, 2025a). While this theoretical foundation is essential, the translation of these mechanisms into systematic training approaches remains insufficiently addressed in the literature (Edwards et al., 2002; Mauger, 2014; Konings and Hettinga, 2018b; Menting et al., 2022).

Recent reviews have highlighted the need for greater integration between training science and pacing behavior, emphasizing how athletes might deliberately develop the capacity to execute advanced pacing strategies under competitive conditions (Edwards et al., 2002; Konings and Hettinga, 2018b; Menting et al., 2022). In this context, negative split pacing should be conceptualized not merely as a race-day tactic, but as a trainable skill emerging from the interaction of physiological capacity, cognitive control, and perceptual regulation. This article aims to bridge this gap by providing evidence-informed, experience-based guidelines to help coaches and athletes systematically develop the capacity to execute negative split strategies in competition. Structured training models, progressive mesocycle designs, and practical monitoring frameworks are presented for both recreational and competitive endurance athletes.

Rather than relying solely on race-day pacing decisions, we propose that the capacity for negative split execution must be deliberately trained through targeted physiological adaptations, pacing awareness, and progressive exposure to controlled discomfort. By shifting the focus from “describing” negative splits to their systematic development, this review emphasizes practical pathways for supporting consistent performance and race-day execution, particularly in the marathon and half-marathon. Accordingly, this mini-review aims to synthesize physiological, perceptual, and behavioral research on pacing regulation to propose an evidence-informed framework for developing negative split pacing capacity in endurance athletes. The proposed framework may also be adapted to other endurance contexts—such as trail running, cycling time trials, or long-distance triathlon—where pacing discipline and fatigue management are critical determinants of success.

## 2 Training foundation for negative split pacing capacity

Negative split pacing should not be viewed as a spontaneous race-day decision, but as a trainable physiological and perceptual capacity that emerges from the interaction of metabolic regulation, fatigue resistance, cognitive control, and environmental tolerance. Successful execution requires athletes to regulate effort conservatively during the early race stages while preserving the capacity to accelerate under increasing physiological strain in the second half. Accordingly, the development of negative split capacity depends on targeted adaptations that support controlled energy expenditure, stable perception of effort, and resistance to central and peripheral fatigue (Grivas, 2025a).

### 2.1 Lactate threshold and metabolic control

A central physiological determinant of negative split pacing is the ability to sustain intensities close to the LT without excessive accumulation of metabolic by-products. Athletes with a higher LT relative to race pace can delay the onset of acidosis and maintain muscular efficiency during late-race surges, enabling a progressive increase in speed as fatigue accumulates. Performance analyses have consistently shown that athletes who avoid early metabolic perturbation and excessive lactate accumulation are more likely to maintain even or negative pacing profiles in endurance events (Foster et al., 1994; Abbiss and Laursen, 2008; Hanley, 2015).

Training interventions targeting the LT have consistently been shown to improve metabolic stability and pacing control in endurance athletes. Sustained tempo runs and cruise intervals performed at or slightly below LT enhance lactate clearance capacity and improve tolerance to prolonged submaximal intensities (Billat, 2001; Helgerud et al., 2007; Seiler, 2010). These adaptations support pacing strategies characterized by restrained early effort and preserved metabolic reserve, thereby facilitating controlled acceleration during the latter stages of competition. From a pacing perspective, LT-focused training expands the margin between sustainable intensity and critical fatigue thresholds, reducing the likelihood of late-race deceleration (Foster et al., 1994; Abbiss and Laursen, 2008)

### 2.2 VO_2_ kinetics and running economy

Beyond metabolic thresholds, the efficiency with which oxygen uptake responds to changes in exercise intensity plays a key role in negative split pacing. Faster VO_2_ kinetics reduce the oxygen deficit during exercise onset and pace transitions, thereby limiting reliance on anaerobic metabolism and preserving energetic reserves for later race phases (Jones and Carter, 2000). Athletes exhibiting faster VO_2_ responses are better able to accommodate pace changes without disproportionate increases in physiological strain, a prerequisite for controlled late-race acceleration.

Similarly, RE reduces the energetic cost of locomotion and is a key determinant of endurance performance (Saunders et al., 2004; Joyner and Coyle, 2008; Barnes and Kilding, 2015). By lowering the oxygen/energy cost at a given speed, including under conditions of accumulated fatigue, improved RE may help athletes sustain or increase late-race speed at a lower relative metabolic demand, thereby supporting more even or negative pacing profiles (Abbiss and Laursen, 2008; Fletcher and MacIntosh, 2017).

Training interventions performed near VO_2max_, particularly high-intensity interval training, have been shown to improve VO_2_ kinetics and aerobic power, while neuromuscular drills such as strides and short hill sprints enhance movement efficiency and force application (Jones and Carter, 2000; Billat, 2001). Collectively, these adaptations facilitate smoother transitions between pacing phases and support late-race accelerations without excessive increases in perceived exertion, thereby reinforcing negative split pacing capacity.

### 2.3 Central fatigue resistance

Negative split pacing also depends on the athlete’s capacity to resist central fatigue, defined as a progressive reduction in voluntary neural drive to the working musculature during prolonged exercise (Gandevia, 2001; Taylor et al., 2006). As endurance events progress, central fatigue contributes to rising perceived exertion and constrains the ability to sustain or increase exercise intensity, even in the presence of residual peripheral capacity (Marcora et al., 2009; Pageaux, 2016). Athletes with greater resistance to central fatigue demonstrate enhanced tolerance to prolonged discomfort and a superior ability to mobilize remaining physiological reserves during the final stages of competition, supporting more controlled late-race pacing and finishing capacity. Training interventions that systematically expose athletes to late-session or back-loaded effort appear to enhance tolerance to central fatigue, thereby reinforcing pacing control under conditions of accumulated perceptual and neural strain (Temesi et al., 2014).

### 2.4 Perceptual regulation and pacing discipline

Effective negative split execution depends critically on perceptual regulation and decision-making throughout the event. Contemporary models conceptualize pacing as an anticipatory, perception-based control process in which athletes continuously integrate internal cues—such as perceived exertion, respiratory strain, and muscular discomfort—with knowledge of the remaining distance to regulate effort and preserve finishing capacity (Tucker, 2009; Tucker and Noakes, 2009). Within this framework, pacing is not a passive consequence of physiological limits but a self-regulated behavior shaped by cognitive control, prior experience, and action–perception coupling (Smits et al., 2014; Micklewright et al., 2017).

Empirical and theoretical work supports the notion that effective pacing relies on learned perceptual calibration. Athletes who successfully regulate pace demonstrate an enhanced ability to interpret internal signals accurately and align perceived effort with task demands, particularly under conditions of fatigue and uncertainty (Mauger, 2014; Smits et al., 2014). Longitudinal and developmental studies further suggest that pacing behavior evolves with practice and is trainable across levels of expertise, reinforcing the view that pacing discipline represents an acquired skill rather than an innate trait (Menting et al., 2022). In this context, the execution of even or negative pacing profiles reflects refined perceptual decision-making as much as physiological capacity.

Experimental evidence also demonstrates that pacing regulation is sensitive to cognitive load and decision-making constraints. Manipulations of feedback availability, task knowledge, or performance expectations have been shown to alter effort distribution and tolerance to discomfort during endurance exercise, highlighting the plasticity of pacing behavior (Mauger et al., 2009; Tucker, 2009). Central to this process is the perception of effort, which functions as an integrative signal guiding anticipatory regulation of exercise intensity in relation to the expected endpoint (Pageaux, 2016).

Importantly, cognitive fatigue represents an additional constraint on pacing regulation. Experimental studies have shown that prior mental fatigue increases perceived exertion at a given workload, impairs endurance performance, and constrains the ability to sustain or increase intensity despite preserved physiological capacity (Marcora et al., 2009; Pageaux, 2016). Conversely, repeated exposure to decision-making under perceptual or cognitive strain has been associated with improved tolerance to discomfort and enhanced effort regulation during prolonged exercise, supporting the concept that cognitive resilience relevant to pacing can be trained (Temesi et al., 2014).

From a training perspective, these findings indicate that negative split capacity can be enhanced not only through physiological conditioning but also through deliberate development of perceptual regulation and pacing autonomy. Selected training sessions may therefore be designed to calibrate rate of perceived exertion (RPE) and strengthen decision-making by manipulating external feedback (e.g., RPE-guided efforts, concealed pace displays, or partial-feedback conditions). Structured post-session debriefing that explicitly links perceived exertion to objective performance outputs further reinforces perceptual learning and pacing skill acquisition (Menting et al., 2022). Collectively, these strategies enhance pacing literacy by strengthening the coupling between perception, decision-making, and performance—an essential cognitive prerequisite for controlled late-race acceleration and successful negative split execution.

### 2.5 Thermoregulatory constraints

Thermoregulatory strain represents a critical constraint on negative split pacing, particularly in warm or humid environments. Elevations in core temperature increase cardiovascular strain, increase metabolic strain, and amplify perceived exertion, collectively limiting the capacity to sustain or increase exercise intensity during the latter stages of endurance events (Nybo et al., 2014; Périard et al., 2015). From a pacing perspective, thermal stress reduces the margin for late-race acceleration by constraining both physiological reserve and perceptual tolerance, often leading to conservative or positive pacing profiles under heat stress (Racinais et al., 2015).

Athletes with superior heat tolerance demonstrate improved cardiovascular stability, reduced thermal discomfort, and enhanced perceptual control, which together support more consistent pacing behavior despite rising thermal load (Périard et al., 2015; Racinais et al., 2015). Experimental and field studies have shown that uncompensable heat stress elevates RPE disproportionately relative to mechanical output, reinforcing the central role of perceptual strain in pacing regulation under hot conditions (Nybo and Nielsen, 2001; Nybo et al., 2014).

**FIGURE 1:**
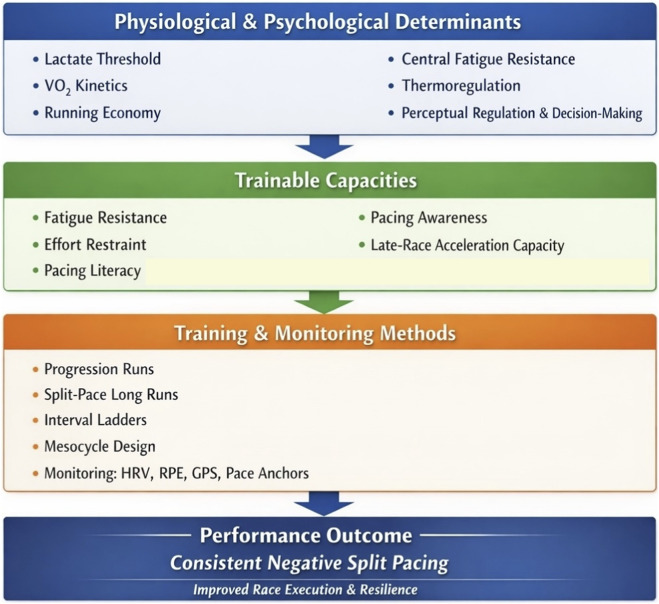
Conceptual framework linking physiological and cognitive mechanisms to pacing literacy, in-race strategy, and performance outcomes in negative split pacing.

Heat acclimation protocols, including repeated training in warm environments or controlled heat exposure, induce well-documented adaptations such as plasma volume expansion, improved sweat rate and distribution, and attenuated perceptual strain (Périard et al., 2015; Racinais et al., 2015). These adaptations improve thermoregulatory efficiency and cardiovascular stability, thereby preserving the athlete’s ability to regulate effort and execute controlled pacing during the latter stages of endurance competition. Consequently, thermoregulatory adaptation constitutes an important, albeit context-dependent, contributor to negative split pacing capacity, particularly in endurance events conducted under environmental heat stress (Figure 1).

## 3 Practical workouts and training blocks

Developing the capacity for negative split pacing requires training stimuli that not only enhance aerobic fitness, but also promote pacing regulation, fatigue tolerance, and controlled exposure to increasing effort in the latter stages of exercise. The endurance training literature consistently emphasizes that pacing behavior is shaped through repeated interaction between physiological strain and perceptual decision-making, rather than emerging spontaneously on race day. Accordingly, specific workout formats have been examined for their ability to elicit adaptations that align with the demands of even and negative split pacing.

Progressive tempo runs are frequently discussed in the literature as an effective means of improving metabolic stability and pacing control. Studies examining threshold-oriented continuous runs indicate that sustained efforts performed below or near the LT enhance lactate clearance capacity, delay the accumulation of fatigue-related metabolites, and improve tolerance to prolonged submaximal intensities (Seiler, 2010; Sandford et al., 2021; Casado et al., 2022). From a pacing perspective, these adaptations reduce the metabolic cost of maintaining a given pace and widen the margin between sustainable intensity and critical fatigue thresholds. This physiological buffer supports the ability to restrain early race effort while preserving sufficient metabolic reserve to increase speed during the latter stages of competition, a defining characteristic of successful negative split execution.

Long-duration aerobic runs incorporating a faster second half, commonly referred to as split-pace or “fast-finish” long runs, have also been examined as a strategy to expose athletes to late-race fatigue while maintaining pacing discipline. Performance analyses and applied studies suggest that such sessions improve glycogen management, neuromuscular efficiency under fatigue, and perceptual tolerance to sustained effort (Hanley, 2015; Sha et al., 2024). By replicating the physiological and psychological conditions encountered in the closing stages of endurance events, these runs facilitate the integration of early restraint with late-race assertiveness. The literature therefore supports their inclusion as a bridge between aerobic conditioning and race-specific pacing demands.

Interval-based formats with progressive or descending intensity structures have been widely employed in endurance training to enhance tolerance to increasing workloads and to refine pacing regulation across variable intensities. Research on interval training indicates that progressively increasing intensity within a session improves aerobic power, neuromuscular recruitment, and the athlete’s capacity to manage rising perceived exertion (Billat, 2001; Abbiss and Laursen, 2008). Experimental work on pacing and effort distribution further suggests that exposure to variable intensity profiles enhances the ability to regulate effort under fatigue and uncertainty (Mauger et al., 2009). Within a negative split development framework, such interval structures provide a controlled environment for rehearsing late-stage acceleration while maintaining perceptual control, thereby strengthening pacing intelligence rather than simply increasing maximal capacity.

Importantly, the literature indicates that these workout types are most effective when implemented within focused training blocks rather than as isolated sessions. Block-based approaches of approximately three to 5 weeks allow athletes to repeatedly engage with similar pacing-related stimuli, promoting consolidation of both physiological adaptations and perceptual learning (Issurin, 2016; Haugen et al., 2022). Early blocks may emphasize threshold stability and metabolic efficiency, while later phases increasingly integrate race-pace specificity and controlled late-stage intensity. A subsequent taper enables the recovery of central and peripheral fatigue while preserving the adaptations necessary for effective pacing execution.

Collectively, the endurance training literature supports the use of progressive tempo runs, split-pace long runs, and progressive interval structures as evidence-informed tools for developing negative split pacing capacity (Abbiss and Laursen, 2008; Seiler, 2010; Sandford et al., 2021). These sessions do not merely increase fitness; rather, they condition athletes to integrate early restraint, fatigue tolerance, and late-race acceleration through repeated, structured exposure to controlled pacing demands. When framed within coherent training blocks and supported by appropriate monitoring, such workouts represent a practical extension of the physiological and perceptual mechanisms underpinning successful negative split performance (Billat et al., 2001; Haugen et al., 2022).

## 4 Weekly and mesocycle planning

The development of negative split pacing capacity requires deliberate weekly and mesocycle planning that integrates physiological adaptation, perceptual skill acquisition, and fatigue management. Rather than emerging spontaneously on race day, the ability to accelerate in the second half of an endurance event appears to depend on repeated, structured exposure to controlled pacing demands embedded within coherent training cycles.

### 4.1 Mesocycle structure and intensity distribution

Across the endurance training literature, mesocycles of approximately 4–6 weeks are commonly used to target specific performance adaptations (Issurin, 2016). In the context of negative split pacing, such mesocycles typically aim to improve LT, VO_2_ kinetics, neuromuscular fatigue resistance, and perceptual control—factors consistently associated with even or negative pacing profiles in endurance competition (Abbiss and Laursen, 2008; Hanley, 2015).

Intensity distribution within the mesocycle plays a central role in shaping these adaptations. Seiler (2010) proposed that endurance performance is optimized through a polarized distribution, characterized by a high volume of low-intensity training combined with a smaller proportion of high-intensity work. More recent evidence suggests that pyramidal distributions, incorporating substantial low-intensity volume alongside moderate- and high-intensity training, may be particularly effective during pre-competition phases (Filipas et al., 2022; Sperlich et al., 2023). From a pacing perspective, such distributions allow athletes to accumulate aerobic capacity while selectively targeting intensities relevant to late-race acceleration.

Importantly, mesocycle planning that progressively increases exposure to race-relevant intensities appears to support the development of pacing restraint early in exercise and controlled acceleration under fatigue. Accordingly, Table 1 synthesizes an evidence-informed 4-week mesocycle structure derived from the endurance training literature, aligning intensity distribution with key physiological and perceptual targets relevant to negative split execution (Table 1).

**TABLE 1:** Example of a 4-week mesocycle for developing negative split capacity in endurance athletes.

Week	Focus	Key session	Notes
1	Aerobic base and pacing control	10 km progressive tempo run (last 3 km at ∼90–95% vLT pace)	Emphasize pace awareness, RPE ∼3-4
2	Lactate threshold development	4 × 2 km intervals with pace progression (90%–100% vLT)	Negative split within reps; short recovery (2 min)
3	Long run simulation	26 km long run with final 8 km at target race pace	Mimics fatigue-resistance and finishing power
4	Taper and race preparation	50% volume reduction, 2 short workouts at race pace	Maintain intensity, sharpen, reduce fatigue

LT: lactate threshold; RPE: rating of perceived exertion; vLT: velocity lactate threshold.

### 4.2 Weekly organization, recovery, and tapering

The development of negative split pacing capacity depends not only on the selection of appropriate training stimuli but also on how these stimuli are organized within the weekly microcycle and supported by adequate recovery. Endurance training literature consistently highlights that the ability to accelerate late in exercise is strongly influenced by the management of cumulative fatigue across the training week, particularly central and neuromuscular fatigue (Seiler, 2010; Issurin, 2016).

From a weekly organization perspective, alternating demanding sessions that target race-relevant intensities with low-intensity recovery days appears essential for preserving pacing control. Studies examining training intensity distribution indicate that clustering high-intensity or threshold-oriented sessions without sufficient recovery impairs effort regulation and increases the likelihood of early overexertion during subsequent sessions (Seiler, 2010; Filipas et al., 2022). Conversely, distributing key workouts across the week with adequate low-intensity volume supports both physiological adaptation and perceptual freshness, facilitating controlled effort early and the capacity to increase pace under fatigue.

Recovery plays a central role in maintaining the neuromuscular and perceptual readiness required for negative split execution. Monitoring approaches based on internal load markers, such as session RPE, heart rate variability (HRV), and subjective recovery indices, have been shown to inform day-to-day training adjustments and reduce the risk of non-functional overreaching in endurance athletes (Plews et al., 2012; Manresa-Rocamora et al., 2021). From a pacing perspective, preserving autonomic balance and perceptual sensitivity is particularly important, as accumulated fatigue can distort effort perception and compromise decision-making during the early stages of exercise, a phase where premature pacing errors are most likely to occur in endurance events.

The tapering phase represents a critical period for consolidating adaptations related to negative split pacing. Meta-analytic evidence indicates that reductions in training volume of approximately 40%–60%, while maintaining intensity, optimize performance outcomes by alleviating both central and peripheral fatigue without inducing detraining (Bosquet et al., 2007). Importantly, maintaining exposure to race-pace or slightly faster efforts during the taper reinforces confidence in late-race acceleration and supports the retention of pacing discipline developed during the preceding mesocycle.

Collectively, effective weekly organization, recovery management, and tapering strategies provide the structural conditions under which negative split pacing capacity can be expressed in competition. When aligned with the physiological and perceptual adaptations developed earlier in the training cycle, these planning principles support the transition from training-based pacing control to reliable race-day execution (Bosquet et al., 2007; Seiler, 2010; Issurin, 2016). Table 2 presents a practical 6-week example of weekly organization, recovery management, and tapering designed to support negative split pacing capacity (Table 2).

**TABLE 2:** Six-week mesocycle for developing negative split pacing capacity.

Week	Primary focus	Key workouts	Monitoring and feedback
1	LT calibration and aerobic efficiency	2 × tempo runs (20–25 min at LT), long run 90 min easy	HRV baseline, RPE awareness
2	Controlled fatigue and pacing awareness	1 progression run (start easy → finish LT), 1 interval session (6 × 3 min at 95%–100% LT)	HR drift <5%, RPE <7 early, <8 late
3	Endurance and resistance to central fatigue	Split-pace long run (120 min with final 20 min at goal pace), 1 hill sprint session	Post-run reflection on pacing and control
4	Pacing literacy under fatigue	Back-loaded intervals (5 × 1 km at race pace, last 2 faster), 1 progressive tempo	RPE progression across session, pace variability <3%
5	Race-specific preparation	Marathon simulation (25 km at 90%–100% goal pace), 1 recovery run	GPS + RPE correlation, perceived readiness
6	Taper and race rehearsal	Tune-up run (10 km at race pace with last 3 km negative split), volume ↓ 50%	HRV recovery trend, freshness index

LT: lactate threshold; HRV: heart rate variability; RPE: rating of perceived exertion; HR: heart rate.

## 5 Evidence for negative split pacing success in competition

Performance analyses consistently indicate that even and negative split pacing profiles are associated with superior outcomes in long-distance endurance events, particularly among faster and more experienced athletes (March et al., 2011; Deaner et al., 2015; Hanley, 2015). These studies suggest that successful performance is less dependent on aggressive early pacing and more strongly related to the ability to regulate effort, limit early fatigue accumulation, and preserve physiological and perceptual reserves for the latter stages of competition (March et al., 2011; Deaner et al., 2015; Hanley, 2015).

Within elite endurance running, several high-profile performances provide illustrative examples of this pacing control. Eliud Kipchoge’s 1:59:40 performance during the INEOS 1:59 Challenge was characterized by exceptionally even pacing, with kilometer splits maintained within a narrow range (2:48–2:52 min km^-1^), reflecting precise effort regulation and optimized energy distribution across the entire distance (INEOS 1:59 Challenge, 2019). In contrast, pronounced negative split strategies were observed in Kenenisa Bekele’s 2019 Berlin Marathon performance and Kelvin Kiptum’s 2023 marathon world record, where substantial acceleration occurred in the second half of the race, highlighting the capacity to mobilize remaining physiological reserves under severe fatigue (Ramsak, 2019; McAlister, 2023; Grivas, 2025b). These performances exemplify different expressions of pacing mastery—either through extreme even pacing or controlled late-race acceleration.

Beyond elite competition, large-scale observational studies provide important insight into pacing strategy effectiveness across performance levels. Hanley (2015) demonstrated that faster finishers at the IAAF World Championships were more likely to adopt even or negative pacing profiles, whereas slower athletes exhibited greater positive splits and late-race deceleration. Similarly, analyses of millions of marathon performances show that recreational runners frequently experience substantial pace deterioration in the final stages, a phenomenon commonly described as “hitting the wall” (Smyth, 2021). This pacing collapse is particularly prevalent among less experienced athletes and is associated with considerable performance loss relative to predicted outcomes.

Collectively, the competition and performance-analysis literature suggests that while successful negative split pacing is relatively rare outside elite cohorts, athletes who minimize early overexertion and maintain pace stability consistently achieve superior performance outcomes, supporting the feasibility and value of negative split pacing as a trainable strategy across performance levels. Table 3 synthesizes evidence from key performance analyses across elite, sub-elite, and recreational cohorts, highlighting consistent associations between pacing stability, late-race performance, and competitive success (Table 3).

**TABLE 3:** Summary of evidence on pacing strategy outcomes across performance levels in endurance running.

Study	Sample/Event	Performance level	Main pacing findings	Implications for negative split pacing
March et al. (2011)	Large marathon datasets	Recreational–sub-elite	Faster runners showed smaller pace decrements after halfway; slower runners exhibited pronounced late-race slowing	Early pacing restraint is associated with superior overall performance
Deaner et al. (2015)	Marathon runners	Recreational	Better performers paced more evenly with lower pace variability	Even pacing reflects superior pacing skill and experience
Hanley (2015)	IAAF world championship marathons	Elite	Top finishers more frequently adopted even or negative split profiles	Negative split pacing is feasible and advantageous at the elite level
Abbiss and Laursen, (2008)	Review of pacing strategies	Mixed	Even and negative pacing profiles linked to optimal endurance performance	Theoretical support for negative split pacing as an optimal strategy
Smyth (2021)	>4 million marathon performances	Recreational	Majority experienced late-race pacing collapse (‘hitting the wall')	Highlights difficulty but performance value of negative split pacing
Renfree and St Clair Gibson (2013)	Competitive endurance events	Trained	Successful athletes regulated pace proactively rather than reactively	Supports pacing as a learned, anticipatory skill
Tucker et al. (2006)	Track endurance events	Elite	Positive pacing in short events; longer events favor even/negative pacing	Distance-dependent feasibility of negative split strategies
Konings and Hettinga (2018a)	Review of pacing regulation	Mixed	Pacing shaped by physiology, perception, and tactical context	Negative split pacing depends on fitness and decision-making
Casado et al. (2022)	Elite distance runners	Elite	High-level runners showed superior pace stability and late-race control	Training status underpins negative split capacity

## 6 Monitoring and feedback

Effective monitoring plays a central role in developing the physiological and perceptual control required for successful negative split execution in endurance racing. Advances in wearable technology, including GPS watches, heart rate monitors, power meters, and AI-driven training platforms, have enabled athletes and coaches to access a wide array of real-time physiological and performance metrics. These tools facilitate dynamic regulation of pacing and intensity throughout both training and competition. HRV, for instance, can be used to track recovery status and readiness to train (Plews et al., 2012), while running power and LT estimations offer insight into effort sustainability. Modern wearables can also estimate VO_2_ kinetics and fatigue accumulation, allowing athletes to avoid premature deceleration and to preserve metabolic resources for a stronger race finish (Bourdon et al., 2017).

Beyond physiological feedback, perceptual cues, such as RPE, remain essential for pacing regulation, especially under fatigue or in unpredictable race environments. Experienced endurance athletes tend to display superior internal regulation through conscious modulation of effort based on RPE, often described as a form of “teleoanticipation” (Tucker, 2009). This refers to the cognitive preplanning of effort distribution relative to the perceived distance remaining. During a negative split strategy, athletes typically experience a progressive increase in RPE across race stages, without crossing the critical fatigue threshold too early (Mauger et al., 2009; Brick et al., 2014).

Recent developments in artificial intelligence and personalized feedback systems have also enhanced the accuracy and accessibility of pacing guidance for non-elite runners. Tools that integrate real-time data with predictive modeling can inform adaptive training plans, optimize tapering, and provide in-race alerts to ensure adherence to pacing targets. Such technology can support not only elite athletes but also recreational runners aiming to implement negative splits more systematically (Grivas and Safari, 2025).

Taken together, these monitoring strategies, physiological, perceptual, and technological, form a comprehensive framework for supporting pacing discipline, reducing pacing variability, and enhancing the probability of executing a successful negative split under both training and race conditions. Table 4 synthesizes key physiological targets, corresponding training modalities, and monitoring tools supported by the reviewed literature (Table 4).

**TABLE 4:** Key physiological targets, training modalities, and monitoring tools for negative split capacity.

Targeted adaptation	Training modality	Monitoring/Feedback tools
LT optimization	Tempo runs, progression runs, split-pace intervals	Heart rate monitor, blood lactate (if available), perceived exertion
VO_2_ kinetics and oxygen utilization	Intervals at vVO_2max_, short recoveries	VO_2_ monitoring (lab), HR drift, RPE scale
Central fatigue resistance	Long runs with a fast finish, back-to-back long runs	RPE, HR variability, and neuromuscular fatigue indicators
Pacing awareness and decision-making	Progressive runs, negative split simulations	GPS watch, pace/km monitoring, in-run feedback
Psychological toughness/pacing control	Time-based intervals under fatigue, split ladders	RPE, subjective readiness, session-RPE
Recovery and load management	Periodized cycles, tapering blocks	HRV, TSB, wearable readiness scores

LT: lactate threshold; RPE: rating of perceived exertion; HRV: heart rate variability; TSB: training stress balance; HR: heart rate.

## 7 Practical recommendations for coaches: translating negative split pacing theory into training

Designing training programs to support negative split pacing strategies requires coaches to balance physiological development, perceptual learning, and race-day discipline. While elite runners may already possess the neuromuscular and metabolic prerequisites to sustain such strategies (Foster et al., 1994; Abbiss and Laursen, 2008), the ability to execute them consistently must be cultivated over time through structured and intentional practice (Mauger, 2014).

For non-elite athletes, developing negative-split capacity hinges on repeated exposure to workouts that simulate progressive pacing. The literature suggests that the inclusion of tempo runs that conclude at a higher relative intensity than they begin, split-pace long runs incorporating a faster terminal segment at or near race pace, and interval sessions with descending split patterns may be beneficial for developing negative split pacing capacity (Seiler and Tønnessen, 2009). These training formats have been shown to enhance LT-related regulation, fatigue resistance, and the capacity to delay early effort expenditure, thereby preserving the ability to increase or maintain intensity during the later stages of exercise (Jones and Burnley, 2009).

Over a 4–6 weeks mesocycle, progression-based workouts can be structured with gradually increasing intensity in the second half, aligned with the athlete’s race goals and fitness level. The training load should be carefully monitored through both external (e.g., pace, distance, duration) and internal (e.g., heart rate, RPE) measures, ensuring adaptation without overreaching (Bourdon et al., 2017). In the taper phase, race-pace intervals and finish-strong sessions should reinforce confidence in late-race acceleration (Bosquet et al., 2007).

Monitoring tools such as GPS watches and heart rate monitors have been shown to provide valuable feedback on pacing patterns, cardiac drift, and training intensity distribution, thereby supporting pacing regulation and training individualization (Plews et al., 2013; Bourdon et al., 2017). When integrated with perceptual markers like the session RPE (Foster et al., 2001), they help athletes become more attuned to effort regulation, a key determinant of negative split success (Smirmaul and de, 2012). Table 5 synthesizes common errors observed during negative split attempts, their underlying causes, and evidence-informed practical corrections derived from coaching practice and the pacing literature (Table 5).

**TABLE 5:** Common errors in negative split attempts and practical corrections.

Common error	Underlying cause	Practical correction
Starting too fast	Adrenaline, overestimation of fitness	Controlled first 5 km, set pace alerts on GPS watch
Inability to accelerate in the second half	Inadequate endurance or glycogen depletion	Incorporate long runs with a fast finish; train fueling strategy
Misjudging LT	Lack of physiological awareness	Use tempo runs and HR monitoring to refine pace perception
Over-reliance on external pacing	Inexperience or group pacing pressure	Practice solo negative split runs to develop internal regulation
Poor fatigue resistance in the final 10 km	Insufficient long-run stimulus	Back-to-back long runs or split-paced simulation efforts
Inconsistent pacing due to terrain or weather	Lack of terrain-specific training	Include hill training or simulation in varied conditions
Ignoring perceptual cues (RPE, breathing, etc.)	Focus only on numbers (pace/HR)	Integrate subjective feedback (RPE log, post-run reflections)
Inadequate taper before race	Fear of losing fitness or poor periodization	Structured taper with reduced volume, maintained intensity

LT: lactate threshold; RPE: rating of perceived exertion; HR: heart rate.

Psychological training should also be embedded throughout the preparation process. Teaching athletes to delay gratification, resist early race surges, and trust a pre-defined pacing plan are central elements of successful execution (Brick et al., 2014). Cognitive strategies such as self-talk, visualization of late-race surges, and attentional focusing can enhance control under fatigue and increase resistance to “panic pacing” in the early stages (McCormick et al., 2015).

Importantly, feedback loops between athlete and coach are essential. Structured debriefs after key workouts and races should analyze both pacing data and subjective experience, reinforcing the relationship between perception and performance. Adjustments can then be made to refine strategy and workload across the training cycle (Stellingwerf, 2012).

Ultimately, coaching for negative split pacing is not solely about optimizing physiology. It involves fostering a pacing literacy that allows athletes to make real-time decisions based on bodily feedback, external conditions, and race dynamics. This blend of physiological capacity and cognitive control, when systematically trained, can transform negative split pacing from an abstract theory into an executable skill across all levels of endurance performance.

## 8 Critical perspectives and future directions

Despite growing interest in pacing strategies, the current evidence base linking specific training interventions to successful negative split execution remains largely indirect. Much of the available literature is observational or cross-sectional, relying on performance analyses rather than longitudinal interventions. As a result, causal relationships between individual physiological or perceptual adaptations and negative split pacing capacity cannot yet be firmly established.

In addition, the feasibility of negative split pacing is highly context-dependent. Tactical race dynamics, environmental stressors (e.g., heat or altitude), and inter-individual differences in physiology, experience, and risk tolerance can substantially constrain pacing behavior. In championship settings, pacing decisions may be driven more by opponent behavior than by pre-planned strategies, while in recreational runners, limited experience or overambitious early pacing often overrides physiological preparedness. These contextual factors highlight that negative split pacing should be viewed as an adaptable framework rather than a universally applicable prescription.

As a mini-review, the present work synthesizes selected theoretical, empirical, and applied literature rather than providing a systematic or exhaustive evaluation of all available evidence. The models presented therefore represent interpretive, evidence-informed frameworks that integrate current physiological, perceptual, and applied insights. Their purpose is not to assert definitive causal mechanisms, but to guide applied practice and stimulate future hypothesis-driven and experimental research.

Future research should prioritize longitudinal intervention studies that directly examine the development of pacing skills. Specifically, studies comparing different training models (e.g., feedback-restricted vs. feedback-rich training, or physiological vs. perceptual skill–focused interventions) are needed to determine their relative effectiveness for pacing skill acquisition. Further work should also explore individual variability in pacing learning, the role of cognitive fatigue and decision-making training, and how emerging technologies can support adaptive pacing under real-world race conditions.

## 9 Conclusion

Negative split pacing emerges from the present synthesis as a multifaceted, trainable performance skill rather than a race-day heuristic or an exclusive attribute of elite athletes. The collective evidence reviewed indicates that its successful execution depends on the interaction of key physiological determinants (LT regulation, VO_2_ kinetics, RE, thermoregulatory efficiency), resistance to central and perceptual fatigue, and advanced perceptual–cognitive regulation of effort.

Across the reviewed literature, athletes who demonstrate superior negative split capacity consistently exhibit the ability to conserve metabolic and perceptual resources early, maintain effort stability under accumulating fatigue, and selectively mobilize physiological reserve during the latter stages of competition. Importantly, these characteristics appear responsive to systematic training interventions grounded in established endurance training principles, including progressive overload, specificity, structured exposure to late-stage fatigue, and feedback-informed perceptual learning.

From an applied perspective, the synthesis of physiological, behavioral, and performance-analysis evidence supports the deliberate use of progression runs, split-pace long runs, and progressively structured interval formats within coherent mesocycle designs. When combined with individualized monitoring strategies—such as session RPE, heart rate variability, and wearable-derived pacing feedback—these methods provide a practical pathway for developing pacing literacy and enhancing race-day execution.

The primary contribution of this review lies in integrating disparate strands of pacing research into a unified, evidence-informed framework for negative split pacing development. By shifting the focus from descriptive accounts of elite performances to a trainable model of pacing skill acquisition, this work offers coaches and practitioners a structured roadmap for translating pacing theory into applied training practice. As endurance sport continues to incorporate advances in monitoring technology and decision-support systems, such integrative frameworks may play an increasingly central role in optimizing performance consistency and late-race resilience across competitive levels.
